# Piezoelectric-Based Vibration Energy-Harvesting for Bladed Disks: Modeling and Comparative Performance Analysis of Interface Circuits

**DOI:** 10.3390/s26113496

**Published:** 2026-06-01

**Authors:** Fengling Zhang, Lve Wang, Tiechun Ding

**Affiliations:** School of Aeroengine, Shenyang Aerospace University, Shenyang 110136, China; wanglue1@stu.sau.edu.cn (L.W.); c4h9feoh@163.com (T.D.)

**Keywords:** blisk, piezoelectric, vibration energy harvesting, interface circuit, synchronized switch

## Abstract

Focusing on the self-powering demand of aircraft engine bladed disks (blisks), this paper investigates piezoelectric vibration energy-harvesting modeling and non-linear circuit performance. A multi-sector electromechanical coupled model is established to analyze the frequency splitting and vibration localization induced by minor structural mistuning. By breaking the cyclic symmetry, mistuning severely concentrates vibration energy into a specific sector, providing a localized high-energy concentration region for optimal energy extraction. To enhance recovery efficiency and load adaptability, three interface circuit topologies—Standard Energy-Harvesting (SEH), Parallel Synchronized Switch Harvesting on Inductor (P-SSHI), and Double Synchronized Switch Harvesting (D-SSHI)—are comparatively analyzed. Through wideband spatial–spectral dynamic response and steady-state impedance matching analyses, the non-linear energy conversion and transfer mechanisms are systematically characterized. Results demonstrate that synchronized switching circuits significantly improve energy transmission via forced voltage inversion, accompanied by a notable equivalent stiffness enhancement effect induced by electromechanical coupling. Furthermore, the D-SSHI topology not only exhibits substantial advantages in peak power extraction, but also, owing to its internal LC energy decoupling mechanism, forms a broad load-independent power plateau across an extremely wide impedance range. This research provides robust theoretical foundations for designing highly resilient self-powered intelligent blades under extreme operating conditions.

## 1. Introduction

In recent years, piezoelectric smart structures have received widespread attention in the field of vibration control for aircraft engine blades. From the perspective of active vibration control, Min [1] utilized piezoelectric actuators to reduce the ideal vibration level of blades, providing a novel approach for achieving thinner blade designs and mitigating fatigue damage. Duffy [2] strategically deployed thin piezoelectric elements in the high-resonant-strain regions of composite blades, achieving effective control over blade vibration. Bendine [3] employed surface-mounted piezoelectric Macro Fiber Composite (MFC) patches to conduct experimental and numerical analyses of the vibration control of fan blades. These studies indicate that strategically deploying piezoelectric elements at critical locations is of great significance for enhancing the vibration reduction performance and service reliability of composite blades.

The realization of piezoelectric smart blades not only relies on vibration control methods but also imposes higher requirements on blade geometry reconstruction and structural modeling accuracy. Wang [4] combined surface fitting algorithms with 3D modeling technology to reconstruct surfaces from blade point cloud data, verifying the reconstruction accuracy through a 3D comparison with the original model. The shape reconstruction method, based on the binocular vision proposed by Qiu [5], significantly improved the surface fitting accuracy of flexible structures. From the perspectives of laser scanning reconstruction and point cloud preprocessing, respectively, Sun and Hu [6] improved the quality of the 3D reconstruction of blade profiles by refining algorithms, laying the foundation for the subsequent layout optimization of piezoelectric sensors and actuators.

During the modeling process of piezoelectric smart structures, hysteresis nonlinearity is a critical factor affecting system accuracy and control performance. The traditional Bouc–Wen model can describe symmetric hysteresis characteristics but struggles to characterize asymmetric and rate-dependent behaviors. To address this, Zhu [7] and Gan [8] improved the model by introducing asymmetric factorial terms and input voltage frequencies, respectively. Qian [9] constructed a hybrid model capable of describing rate-dependent hysteresis characteristics by combining the least squares method with the Bouc–Wen model. Li [10] further proposed a combined Hammerstein–Wiener model. Building on this, Yang [11] established a fractional-order-based rate-dependent Hammerstein model, thereby more accurately describing the dynamic and hysteretic characteristics of piezoelectric actuators. Aiming at the vibration response amplification problem in mistuned-blisks of aircraft engines, the research focus has gradually shifted towards semi-active and passive piezoelectric vibration reduction technologies. Zhang [12] introduced a double-beam system with SSDI-net, which was simplified to a lumped parameter electromechanical coupling model and analyzed using the multi-harmonic balance method with alternating frequency–time techniques (MHBM/AFT). Zhang [13] introduced an enhanced SSDI approach to suppress the vibration of bladed disks in aero-engines.

Research on piezoelectric vibration energy-harvesting interface circuits, which are closely related to the aforementioned synchronized switch damping technologies, has also made significant progress. In recent years, piezoelectric vibration energy-harvesting technology has shown immense potential in fields such as the Internet of Things (IoT), wireless sensor networks, and wearable devices, and the optimal design of interface circuits remains the core challenge in enhancing its conversion efficiency. Traditional rectifier circuits suffer from low efficiency under low-frequency and weak excitation environments. Consequently, synchronized switch harvesting on inductor/capacitor interfaces (SSHI and its variants), akin to SSDI circuits, has become a research hotspot. These circuits achieve nonlinear charge extraction and voltage inversion through synchronized switching actions at the extrema of voltage/displacement, which can significantly increase output power. Yang et al. [14] reviewed interface circuits for small-scale devices, primarily evaluating the practicality of switch-inductor topologies like SSHI and SSDI; Al Ghazi et al. [15] systematically summarized the latest advancements in self-powered piezoelectric energy-harvesting interfaces, comparing the efficiency enhancements of various synchronized switching technologies via tables; Zhang et al. [16] proposed an optimized self-powered parallel SSHI (P-SSHI) interface, experimentally verifying a 1.16- to 2.42-fold increase in output power compared to traditional circuits; Hoseyn et al. [17] integrated SSHI into a thin-plate multi-modal piezoelectric harvesting system, achieving highly efficient multi-modal energy extraction; Ben Ammar et al. [18] developed a self-powered P-SSHI circuit specifically for low-frequency scenarios with irregular foot impact excitations; Wang et al. [19] designed a thermoelectric-assisted, scalable, self-powered P-SSHI array interface supporting the collaborative harvesting of multiple piezoelectric units. Another study [20] proposed an ultra-low-power intermittent control SSHI interface suitable for micro-power embedded systems; Liu et al. [21] innovated a multiple-input MI-SSHI circuit, expanding the multi-source adaptability of the synchronized switch inductor topology; Yang et al. [22] combined SSHI with impedance matching technology to construct a highly efficient energy-management circuit, further improving the performance of shock-type harvesting; Wang et al. [23] developed a fully autonomous SSHIC interface supporting configurable multi-step bias flipping, achieving integrated high-performance harvesting; Du et al. [24] proposed a fully integrated split-electrode SSHC rectifier (a variant of SSHI) applicable to a wide load range; Xia et al. [25] designed a self-powered voltage-multiplying Series-SSHI (S-SSHI) circuit, eliminating the traditional rectifier stage; Wang et al. [26] realized a 0.24 mm^2^ bridgeless hybrid SSHI interface, achieving up to a 1620% enhancement in power extraction. Subsequent research [27] explored networked SSHI configurations for plate-like structures. A conference paper [28] demonstrated a scalable parallel synchronized switch interface for large-scale piezoelectric arrays; another work [29] focused on optimizing the self-powered P-SSHI in vibration environments. Amri et al. [30] further enhanced the nonlinear efficiency of piezoelectric energy-harvesting through SSHI technology optimization. A 2022 IEEE ICIEA conference paper [31] proposed a multi-input P-SSHI interface supporting arbitrary phase differences. Recent work [32] developed a Synchronized Switch Harvesting on Capacitor (SSHC)-integrated circuit as a capacitor-enhanced variant of SSHI/SSDI, while Wang et al. [33] designed an SSHCI hybrid circuit, merging the advantages of both to achieve higher charge extraction efficiency.

Although the aforementioned literature indicates that synchronized switching interface circuits have made significant progress, previous studies mostly evaluated these non-linear circuits on simplified single-degree-of-freedom structures. In the context of rotating multi-sector structures, existing research mainly focuses on vibration suppression, viewing structural mistuning as a negative factor. The integration of structural dynamics in mistuned aero-engine blisks with the non-linear energy extraction characteristics of synchronized switching circuits requires further investigation.

To bridge these research gaps, the major innovations and contributions of this research are summarized as follows:

(1) Unlike studies that focus on vibration suppression, this work utilizes the vibration localization effect induced by structural mistuning to concentrate mechanical energy into a specific sector, providing a targeted region for energy extraction.

(2) A multi-degrees-of-freedom dynamic model is established to systematically evaluate non-linear switching circuits (SEH, P-SSHI, DSSH). This model theoretically quantifies the equivalent stiffness strengthening effect caused by electromechanical coupling during synchronized switching actions.

(3) Through steady-state impedance matching analysis, it is demonstrated that the DSSH topology maintains a load-independent power plateau across a wide resistance range. This energy-decoupling mechanism addresses the impedance matching requirements under fluctuating operating conditions.

## 2. Principle of Piezoelectric Energy-Harvesting for Blisks

The blisk structure, equipped with piezoelectric energy harvesters. consists of three main components:

(1) A mechanical structure that captures the kinetic energy of parasitic mechanical vibrations;
(2) Piezoelectric transducers that convert mechanical energy into electrical energy;
(3) An electrical domain for energy storage or power supply.

For each sector of the blisk, two blade modes and one disk mode are retained, such that each sector is modeled as a 3-DOF lumped-parameter system. The entire system consists of N identical sectors. The dynamic characteristics of each sector are determined by the equivalent masses and stiffnesses of the respective blade and disk modes, as well as the coupling stiffness between adjacent disk sectors.

**Figure 1:**
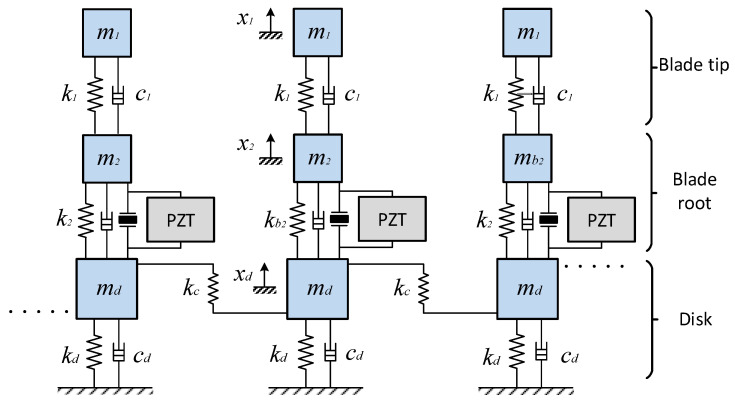
Lumped-parameter model of a blisk with piezoelectric branches.

As illustrated in Figure 1, in this analytical model, the piezoelectric patches are bonded at the blade root. Since the mechanical deformation of the piezoelectric material is strictly driven by the relative displacement between the blade-root DOF ($m_{2}$) and the disk DOF ($m_{d}$), the piezoelectric unit is fundamentally modeled as an electromechanical coupling element bridging these two specific DOFs.

Since the mechanical mass and stiffness of the thin piezoelectric patches are orders of magnitude smaller than those of the massive metallic blisk, their mechanical properties have a negligible impact on the global dynamic characteristics. Furthermore, the operating frequency of the blisk is substantially lower than the intrinsic resonance frequency of the piezoelectric material. Therefore, under a quasi-static assumption, the internal effect and stiffness of the piezoelectric patches are completely disregarded, allowing the mechanical domain to be effectively simplified into a purely structural lumped-parameter model.

To mathematically describe this electromechanical coupling effect, the local behavior of the piezoelectric sensor is first established based on the linear piezoelectric constitutive equations:

(1) $$\left[\begin{array}{l} S\\ D \end{array}\right]=\left[\begin{array}{cc} s^{E} & d_{t}\\ d_{t} & \varepsilon^{T} \end{array}\right]\left[\begin{array}{c} T\\ E \end{array}\right]$$

For linear piezoelectric materials, the vector S denotes the mechanical strain, and the vector T denotes the mechanical stress, while the vectors D and E represent the electric displacement and the electric field intensity, respectively. The elastic compliance matrix $s^{E}$ corresponds to the elastic properties under a constant electric field (short-circuit condition). The $d_{t}$ denotes the piezoelectric coupling coefficients, and $\varepsilon^{T}$ represents the absolute permittivity under constant stress.

Through the spatial integration of these local variables along the bounded piezoelectric patches, a macroscopic lumped-parameter model can be derived. The governing equations of this quasi-static electromechanical conversion can be simplified as follows:

(2) $$\begin{array}{l} F_{pz}=-\alpha e\\ q=\alpha x-C_{pz}e \end{array}$$

Here, $F_{pz}$ denotes the coupling force exerted by the electric field on the mechanical components, x is the mechanical displacement, q is the electric charge, and e is the voltage. The parameter α is the electromechanical coupling coefficient, and $C_{pz}$ is the capacitance of the mechanically unconstrained piezoelectric patch.

Based on these macroscopic relations, a purely structural lumped-parameter model is established for a single sector, characterized by its local mass matrix $M_{s}$, local stiffness matrix $K_{s}$, local damping matrix $C_{s}$ and inter-sector coupling stiffness matrix $K_{c}$.

Ideally, the blisk is periodically symmetric. By defining the kinetic energy T, the elastic potential energy U, and the Rayleigh dissipation function D based on these local sub-matrices, along with the electrical energy of the piezoelectric patches, the extended Lagrange equations can be applied. Consequently, the time-domain governing equations of the fully coupled piezoelectric blisk can be assembled into the following generalized block-matrix form:

(3) $$\left[\begin{array}{cc} M_{\mathrm{ma}} & 0\\ 0 & 0 \end{array}\right]{\ddot{x}}_{p}+\left[\begin{array}{cc} C_{ma} & 0\\ 0 & 0 \end{array}\right]{\dot{x}}_{p}+\left[\begin{array}{cc} K_{ma} & K_{mpp}\\ K_{mpp} & K_{ep} \end{array}\right]x_{p}=f_{p}$$

Here, the mechanical mass, stiffness, and damping matrices of the periodic blisk are constructed using block-circulant operators:

$$\begin{array}{l} K_{a}=\left[\begin{array}{cc} K_{ma} & K_{mpp}\\ K_{mpa}^{T} & K_{ep} \end{array}\right]=\\ \left[\begin{array}{cc} Bcirc(K_{s},K_{c},0,K_{c}^{T}) & Bdiag(K_{mp},\ldots ,K_{mp})\\ Bdiag({K_{mp}}^{T},\ldots ,{K_{mp}}^{T}) & Bdiag(-C_{p},\ldots ,-C_{p}) \end{array}\right] \end{array}$$

(4) $$M_{a}=\left[\begin{array}{cc} M_{ma} & 0\\ 0 & 0 \end{array}\right]=\left[\begin{array}{cc} Bcirc(M_{s},M_{c},\ldots ,M_{c}^{T}) & 0\\ 0 & 0 \end{array}\right]$$

$$C_{a}=\left[\begin{array}{cc} C_{ma} & 0\\ 0 & 0 \end{array}\right]=\left[\begin{array}{cc} Bcirc(C_{s},C_{c},\ldots ,C_{c}^{T}) & 0\\ 0 & 0 \end{array}\right]$$

The subscripts s and c correspond to the internal degrees of freedom within a single sector and the cyclic boundary degrees of freedom, respectively. The global state vector $x_{p}$ is composed of the sector displacements and voltages:

(5) $$x_{a}={\left[x_{1}^{T},\ldots ,x_{i}^{T},\ldots ,x_{\left(N_{s}\right)}^{T},V_{1},\ldots ,V_{i},\ldots ,V_{\left(N_{s}\right)}\right]}^{T}$$

where $N_{s}$ denotes the number of sectors. The vector on the right-hand side consists of the mechanical excitation force $f_{i}$ and the electric charge $Q_{i}$ in each sector:

(6) $$f_{a}={\left[f_{1}^{T},\ldots ,f_{i}^{T},\ldots ,f_{\left(N_{s}\right)}^{T},Q_{1},\ldots ,Q_{i},\ldots ,Q_{\left(N_{s}\right)}\right]}^{T}$$

In realistic turbomachinery environments, the rotating blisk is typically subjected to a traveling-wave aerodynamic excitation. Therefore, assuming the force acts entirely on the blade DOFs, the harmonic excitation force vector $f_{i}$ acting on the i-th sector can be explicitly expressed as:

(7) $$f_{i}(t)=F_{0}e^{j(\omega t-\phi i)}$$

where $F_{0}$ represents the spatial force amplitude vector, and ω denotes the excitation frequency. The inter-blade phase angle is defined as $\phi_{i}=2\pi E(i-1)/N_{S}\phi_{i}$, with E representing the engine order (EO) of the aerodynamic excitation.

With the global electromechanical equations established, interconnecting the piezoelectric network is mathematically equivalent to imposing specific electrical boundary conditions on the electrodes. From the perspective of dynamic equations, a parallel connection between the patches i and j strictly imposes the voltage constraint $V_{i}=V_{j}$. By eliminating the redundant voltage degree of freedom under this constraint, the global electromechanical coupling matrix $K_{mpp}$ and the electrical capacitance matrix $K_{ep}$ are condensed and updated as follows:

(8) $$\begin{array}{l} V=T_{V}V_{r}\\ K_{m}pp^{r}=K_{m}ppT_{v}\\ K_{e}p^{r}=T_{v}^{T}K_{e}pT_{v} \end{array}$$

where V is the original unconstrained voltage vector, and $V_{r}$ is the reduced voltage vector. The transformation matrix $T_{v}$ is a Boolean matrix. For instance, if the i-th and j-th piezoelectric patches are connected in parallel, the constraint $V_{i}=V_{j}$ is applied. Consequently, the j-th column in $T_{v}$ is eliminated, and its non-zero elements are added to the i-th column. This mathematical projection effectively adds the j-th column of $K_{mpp}$ to its i-th column, and sums the corresponding capacitances in $K_{ep}$. Consequently, the redundant voltage variable $V_{j}$ is removed from the state vector, and its corresponding charge $Q_{j}$ is algebraically added to $Q_{i}$ in the excitation vector.

To analytically model the highly nonlinear SSD interconnection between the sub-networks (*i*–*j*) and (*k*–*l*), an equivalent impedance approach based on the first-harmonic assumption is adopted. While this assumption inherently neglects the transient nonlinear voltage clamping in the time domain, it effectively captures the fundamental energy exchange mechanism. Crucially, because the metallic blisk possesses a high mechanical quality factor, it acts as a natural structural low-pass filter, preventing higher-order voltage harmonics from exciting structural resonances. Therefore, this assumption provides highly reliable predictions for the steady-state response while drastically circumventing the computational burden of cumbersome nonlinear time-domain integrations. Accordingly, the frequency-domain governing equations describing the linearized voltage–current relationship of the interconnected SSD are formulated as:

(9) $$\begin{array}{l} \;Q_{k}+Q_{l}=-(Q_{i}+Q_{i})=Q_{int}\\ V_{i}-V_{k}=V_{int}\\ j\omega Z_{SSDI}Q_{int}=V_{int} \end{array}$$

Here, $Q_{int}$ and $V_{int}$ denote the fundamental charge and voltage amplitude across the SSD dipole, respectively. Note that the equations above only govern the parallel interconnection of SSD dipoles; if a cross-interconnection is employed, only the corresponding signs need to be modified. The term $Z_{SSDI}$ denotes the linearized equivalent impedance, given by:

(10) $$Z_{SSDI}(\omega )=\frac{V_{diff\omega }}{I_{diff\omega }}=\frac{\pi (1-\gamma )}{4C_{eq}\omega (1+\gamma )}+\frac{j}{C_{eq}\omega }$$

where the parameter $\gamma =e^{-\pi /2Q_{e}}$ is the electrical attenuation factor related to the circuit quality factor $Q_{e}$. $C_{eq}$ represents the equivalent capacitance of the interconnected network, obtained as the series combination of the individual sub-network capacitances:

(11) $$C_{eq}=\frac{C_{i-j}C_{k-l}}{C_{i-j}+C_{k-l}}$$

Here, $C_{i-j}=C_{k-l}=2C_{p}$ denotes the effective capacitance of each parallel sub-network. Clearly, the equivalent SSD impedance $Z_{SSDI}$ is highly dependent on the vibration frequency ω.

By substituting the equivalent SSD impedance relation back into the condensed dynamic equations, and strictly selecting charge *Q* rather than voltage *V* as the fundamental electrical state variable, the original electromechanical system is transformed. Consequently, the overall equivalent linearized governing differential equation of the blisk coupled with the SSD network is reformulated as:

(12) $$\left[\begin{array}{cc} M_{ma} & 0\\ 0 & 0 \end{array}\right]{\ddot{x}}_{f}+\left[\begin{array}{cc} C_{ma} & 0\\ 0 & C_{ef} \end{array}\right]{\dot{x}}_{f}+\left[\begin{array}{cc} K_{ocf} & K_{cpf}\\ K_{cpf}^{T} & K_{ef} \end{array}\right]x_{f}=f_{f}$$

In Equation (12), the specific sub-matrices are explicitly defined to accommodate the new charge-based state space. First, the right-hand-side generalized excitation vector $f_{f}$ and the equivalent open-circuit structural stiffness matrix $K_{ocf}$ are given by:

(13) $$f_{f}={\left[{f_{1}}^{T},\ldots ,{f_{\left(N_{s}\right)}}^{T},0^{T}\right]}^{T},\;K_{ocf}=K_{m}a+K_{m}ppK_{e}p^{\left(-1\right)}K_{m}pp^{T}$$

To strictly align with the fundamental charge variables, the effective electromechanical coupling matrix $K_{cpf}$ and the global electrical stiffness matrix $K_{ef}$ are mathematically transformed as:

(14) $$K_{cpf}=-K_{mp}∕C_{p},K_{ef}=\left[\begin{array}{cccc} -C_{p} & & & \\ & \ddots & & \\ & & 0 & \\ & & & \ddots \end{array}\right]$$

Most importantly, the equivalent electrical damping matrix $C_{ef}$, which analytically captures the fundamental energy dissipation induced by the nonlinear SSD network, is extracted from the real part of the linearized impedance as:

(15) $$C_{e}f=\left[\begin{array}{cc} 0 & 0\\ 0 & \frac{\pi (1-\gamma )}{4\omega (1+\gamma )C_{eq}} \end{array}\right]$$

Finally, the corresponding global state vector $x_{f}$, which encompasses both the structural displacement degrees of freedom and the reduced independent charge variables, is explicitly defined as:

(16) $$x_{f}={\left[x_{1}^{T},\ldots ,x_{\left(N_{s}\right)}^{T},Q_{1},\ldots ,Q_{int}\ldots Q_{\left(N_{s}\right)}\right]}^{T}$$

Although Equation (12) specifically illustrates the dynamics of a blisk equipped with a single SSD interconnected between sub-networks (*i-j*) and (*k-l*) (with the remaining patches being open-circuited), this theoretical framework can be readily generalized to configurations with multiple SSD networks following the same condensation procedure.

While the baseline structural and electromechanical parameters can be readily extracted using frequency-domain finite element methods with linear circuits, performing transient FEM simulations for structural systems strictly coupled with nonlinear switching circuits is computationally prohibitive due to the immense time-scale disparities. Consequently, adopting the first-harmonic approximation to formulate a purely analytical model is not only supported by the existing literature [34,35] but also practically imperative. The accuracy and reliability of this fully coupled, dimension-reduced theoretical framework will be comprehensively validated against the experimental results presented in Section 5.

## 3. Operating Principle of the Energy-Harvesting Circuit

The blisk dynamic model described above has integrated the SSD (Synchronized Switch Damping) network into the electrical boundary conditions of the piezoelectric patches, thereby achieving a synergy between vibration suppression and energy-harvesting. To further elucidate how the practical circuits realize highly efficient energy extraction on the piezoelectric patches of the blisk, it is necessary to elaborate on the working principles of the energy-harvesting circuits in detail.

The primary limitation of traditional AC-DC energy-harvesting circuits in self-powered energy harvesters (PEH) is that the output current and voltage cannot be maintained in phase, leading to the generation of negative output power. This results in significant losses in the harvested energy.

The Full-Wave Bridge Rectifier (also referred to as Standard Energy-Harvesting, or SEH) is the preferred solution for implementing self-powered energy harvesters. Employing a simple, uncontrolled architecture, it stands as the most widely adopted method in the field of piezoelectric energy-harvesting. When the piezoelectric energy harvester (PEH) generates a positive potential, the full-bridge rectifier converts the voltage into a DC output; conversely, when the PEH generates a negative potential, the voltage is similarly rectified. However, this standard architecture suffers from significant drawbacks, including strong load dependency, low efficiency, and substantial power losses caused by phase misalignment between current and voltage.

To address these limitations, the Synchronized Switch-Harvesting on Inductor (SSHI) technique establishes a resonance by associating an inductor and a switch with the PEH, forming an oscillating LC circuit with the harvester’s internal capacitance. This resonance effect modifies the charge quantity on the parasitic piezoelectric capacitance, thereby maximizing the voltage and enhancing energy extraction efficiency. This technology primarily comprises two interface schemes: Parallel-SSHI (P-SSHI) and Series-SSHI (S-SSHI). The specific classification is determined by the connection of the switch S and inductor L: a series connection implements S-SSHI, whereas a parallel connection implements P-SSHI.

The operating principle of P-SSHI relies on achieving voltage inversion via the control of switch $S_{1}$ and inductor $L_{1}$ following the energy extraction process. During vibration, switch $S_{1}$ remains open, allowing current to flow through the circuit into the storage capacitor. If the voltage of the piezoelectric transducer falls below a specific threshold, switch $S_{1}$ closes automatically, thereby inverting the PEH voltage and terminating the current flow. This implies that the switch remains closed until the complete inversion of the PEH voltage is achieved. Furthermore, the integration of the inductor reduces the phase difference between voltage and current, thereby enhancing energy-harvesting efficiency. However, this voltage inversion generates an electrical damping effect that suppresses the mechanical vibration of the piezoelectric material. This phenomenon, known as Synchronized Switch Damping (SSD), significantly affects the overall conversion efficiency and thus constitutes a major limitation for P-SSHI and S-SSHI circuits.

Lallart et al. proposed another hybrid energy-harvesting scheme, realized by combining the SSHI architecture with the SECE interface. This scheme is named Double Synchronized Switch-Harvesting on Inductor (D-SSHI). Its circuit operating principle is as follows: first, a portion of the harvested energy is transferred from the PEH to an intermediate storage capacitor, while the remaining energy is utilized for the inversion process; subsequently, the energy [in the intermediate storage] is transferred to the inductor and finally delivered to the load.

**Figure 2:**
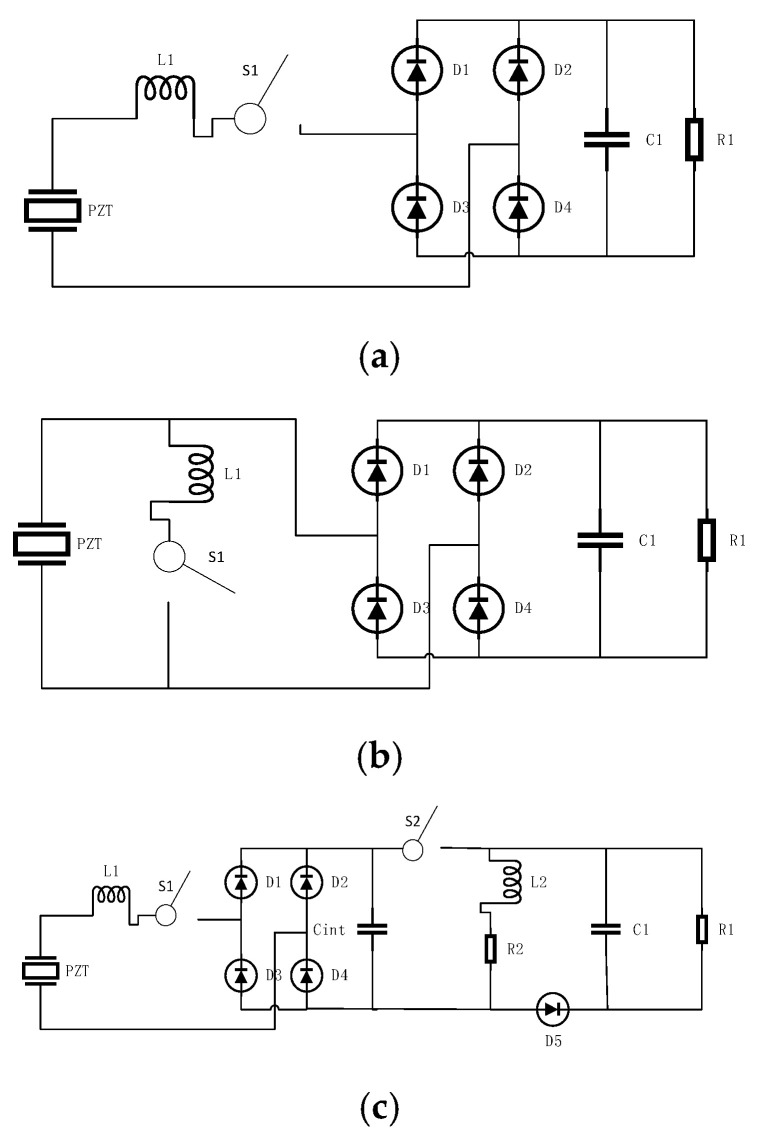
Three different energy switching circuits. (**a**) Series Synchronized Switch-Harvesting on Inductor (S-SSHI) interface; (**b**) Parallel Synchronized Switch-Harvesting on Inductor (P-SSHI) interface; (**c**) Double Synchronized Switch-Harvesting on Inductor (D-SSHI) interface.

Compared to other schemes, D-SSHI exhibits lower sensitivity to load variations due to the indirect connection between the PEH and the load. However, the use of additional components results in the highest energy consumption among the discussed topologies. The circuit involves high complexity due to the double-switch mechanism and is more suitable for high-power application scenarios. The three switching circuits mentioned above are illustrated in Figure 2.

Regarding the Series-SSHI (S-SSHI) interface, when the switch closes to form an LC oscillating circuit, current begins to flow in the piezoelectric patch. When the voltage across the piezoelectric patch reaches zero, the current peaks. After half a vibration cycle, the voltage across the piezoelectric patch inverts. At this point, the charge is transferred to the storage capacitor via the filtering circuit to supply power to the external resistor. When the current drops to zero, the switch opens, and a single energy-harvesting cycle concludes. During operation, the voltage relationship is expressed as follows:

(17) $$\left(V_{m}-V_{DC}\right)=\gamma \left(V_{M}-V_{DC}\right)$$

where γ represents the inversion factor of the synchronized switching circuit, and $V_{DC}$ denotes the voltage across the external load. Under the condition that the displacement amplitude $X_{M}$ of the blisk structure remains constant, the voltage across the load $V_{DC}$ is given by Equation (18)

(18) $$V_{DC}=\frac{2\alpha X_{M}\omega R_{L}\left(1-\gamma \right)}{2R_{L}C_{p}\omega \left(1-\gamma \right)+\pi \left(1-\gamma \right)}$$

Consequently, the harvested power of the S-SSHI energy-harvesting interface is given by

(19) $$P_{S\text{-}SSHI}=\frac{V_{DC}^{2}}{R_{L}}=\frac{4\alpha^{2}X_{M}^{2}\omega^{2}{\left(1-\gamma \right)}^{2}R_{L}}{{\left(2R_{L}C_{P}\omega \left(1-\gamma \right)+\pi \left(1-\gamma \right)\right)}^{2}}$$

Consequently, the harvested power of this interface depends on the external load. Therefore, an optimal resistance exists that enables the interface to achieve maximum harvested power, which is expressed as follows:

(20) $$P_{S\text{-}SSHI\text{-}MAX}=\frac{\alpha^{2}X_{M}^{2}\omega \left(1+\gamma \right)}{2\pi C_{P}\left(1-\gamma \right)}$$

(21) $$R_{L\text{-}opt}=\frac{\pi \left(1-\gamma \right)}{2\pi C_{p}\omega \left(1+\gamma \right)}$$

In a parallel-type synchronized switch inductive (SSHI) energy-harvesting interface, the synchronized switching branch composed of the inductor L and the switch S is connected in parallel with the piezoelectric patch. Therefore, its operating waveform differs from that of the series-type configuration, leading to corresponding changes in both the operating voltage and the harvested power.

When the displacement amplitude of the blisk structure is kept constant, the voltage across the load $V_{L}$ can be expressed as follows:

(22) $$V_{DC}=\frac{2\alpha X_{M}\omega R_{L}}{2R_{L}C_{p}\omega \left(1-\gamma \right)+\pi }$$

The energy-harvesting power can be expressed as follows:

(23) $$P_{P\text{-}SSHI}=\frac{V_{DC}^{2}}{R_{L}}=\frac{4\alpha^{2}X_{M}^{2}\omega^{2}R_{L}}{{\left(R_{L}C_{p}\omega \left(1-\gamma \right)+\pi \right)}^{2}}$$

Similar to the S-SSHI interface, the energy-harvesting power of this interface also depends on the external load. Therefore, there exists an optimal matching resistance that enables the interface to achieve the maximum energy-harvesting power, which is given by:

(24) $$P_{P\text{-}SSHI\text{-}MAX}=\frac{\omega \alpha^{2}{X_{M}}^{2}}{\pi C_{P}(1-\gamma )}$$

(25) $$R_{L\text{-}opt}=\frac{\pi }{C_{p}\omega \left(1-\gamma \right)}$$

The double synchronized switch-harvesting (DSSH) interface can be divided into two parts. The first part consists of the piezoelectric patch, a synchronized switching circuit composed of the inductor $L_{1}$ and the switch $S_{1}$, a diode rectifier, and an intermediate storage (charging) capacitor $C_{\mathrm{int}}$. In this respect, its configuration is essentially the same as that of the previously described S-SSHI energy-harvesting interface.

The second part is a buck–boost converter, which comprises a switch $S_{2}$, an inductor $L_{2}$ (with an internal resistance $R_{2}$), and a diode. This stage transfers the electrical energy stored in the intermediate capacitor $C_{\mathrm{int}}$ to the energy-storage capacitor $C_{1}$ during operation, thereby supplying the load.

Assuming that the vibration displacement amplitude $X_{M}$ of the blisk structure remains constant and that the intermediate capacitor $C_{\mathrm{int}}$ is set to its optimal value, the output power of the DSSH interface can be expressed as follows:

(26) $$P_{DSSH}=\omega \gamma_{c}\frac{{\left(1+\gamma \right)}^{2}\alpha^{2}{X_{M}}^{2}}{2(1-\gamma )C_{P}}$$

## 4. Vibration Energy-Harvesting of Aero-Engine Blisks

### 4.1. Vibration Response Analysis of the Electromechanically Coupled Blisk

To verify the proposed analytical model and investigate the vibration reduction performance, numerical simulations are conducted. The key physical and electrical parameters used in the numerical simulation are determined based on the actual physical parameters of the blisk sector and the piezoelectric patches. The detailed values of these structural and circuit parameters are explicitly listed in Table 1.

**Figure 3:**
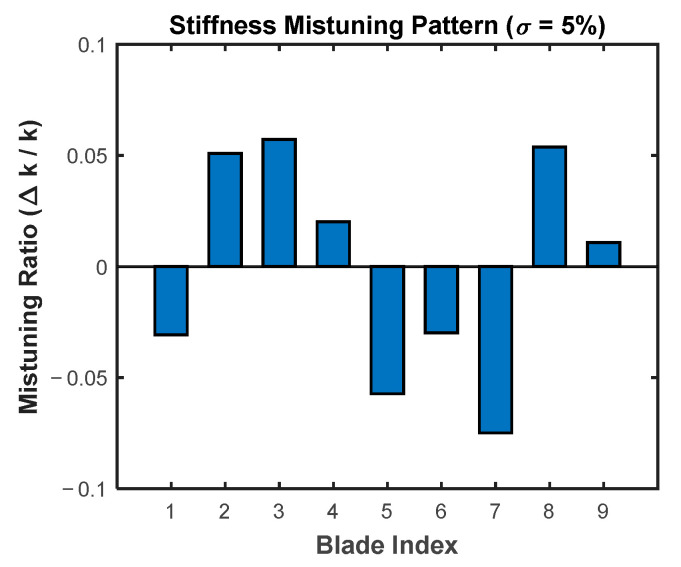
Random Mistuning Patterns.

The mathematical model of the aircraft engine blisk with piezoelectric materials established in Figure 2 consists of nine sectors, each comprising a blade and its corresponding disk portion. The displacement response of the blisk, calculated using the blisk dynamic equation in Equation (12), is illustrated in Figure 3.

Due to various factors, the mistuning phenomenon in blisks is practically unavoidable. Here, stiffness mistuning is assumed, where the individual mistuning parameters are defined as normally distributed random variables with a zero mean and a standard deviation (i.e., the random mistuning strength) of 5%.

**Figure 4:**
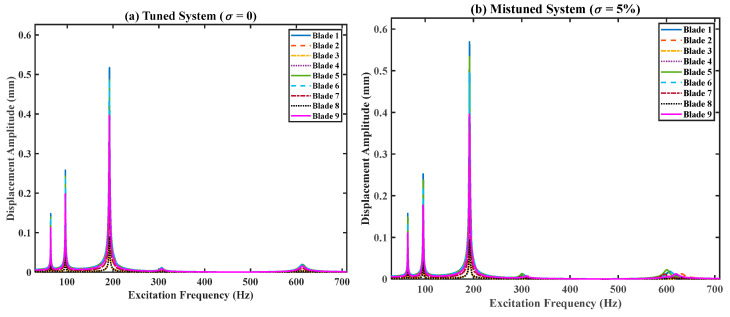
Frequency-Domain Vibration Responses of Tuned and Mistuned Blisks.

Table 2 and Table 3 and the corresponding wideband Frequency Response Function in Figure 4 plots systematically compare the dynamic characteristics of the ideal tuned blisk and the realistic mistuned blisk. Two fundamental physical phenomena induced by mistuning are clearly observed: mode splitting and vibration energy localization.

In the ideal tuned system, the blisk possesses perfect cyclic symmetry. As shown in Table 2, the resonance frequencies of all nine blades in the targeted modal cluster are strictly identical, converging precisely at 192.19 Hz. This symmetry is visually confirmed in the Tuned Frequency Response Function plot, where the responses of all blades coalesce into a single, un-split resonance peak near 192 Hz.

However, upon the introduction of structural mistuning, the cyclic symmetry is broken. Table 1 quantitatively reveals that the identical frequencies split into a scattered frequency band ranging from 191.91 Hz to 192.17 Hz. This frequency splitting phenomenon is particularly conspicuous in the higher-order modal region near 600 Hz of the Mistuned Frequency Response Function plot, where a previously singular peak disperses into multiple distinct, closely spaced resonance peaks.

More importantly, the mistuning triggers severe spatial reallocation of vibration energy. In the tuned state, the maximum displacement of Blade 1 is 0.536 mm. Under the mistuned condition, the vibration energy drastically localizes onto Blade 1, amplifying its peak amplitude to 0.570 mm, representing an amplitude magnification of approximately 6.35%, while simultaneously suppressing the responses of adjacent blades, such as Blade 2, which drops from 0.410 mm to 0.361 mm. As illustrated in the Mistuned Frequency Response Function plot, the highest peak at 192 Hz becomes significantly taller and sharper compared to the tuned baseline.

From an engineering perspective, while this amplitude magnification accelerates high-cycle fatigue in traditional turbomachinery, it provides an exceptional advantage for vibration energy-harvesting. The data robustly proves that Blade 1 acts as the localization hotspot. By intentionally deploying the piezoelectric transducer and the non-linear harvesting interface on this specific sector, the extracted electrical power can be substantially maximized due to the inherently magnified mechanical strain.

### 4.2. Comparative Study on the Parameters of Energy-Harvesting Circuits for the Electromechanically Coupled Blisk

**Figure 5:**
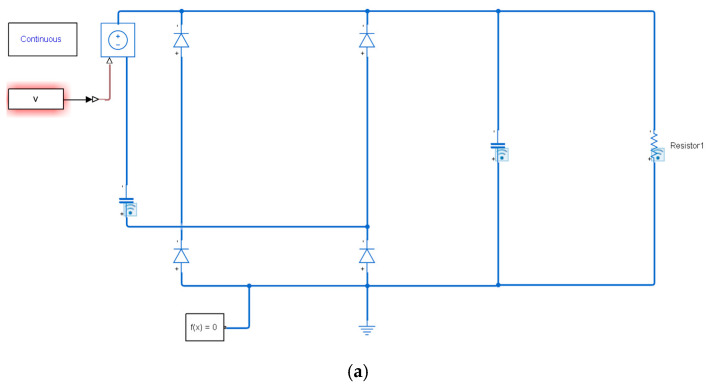

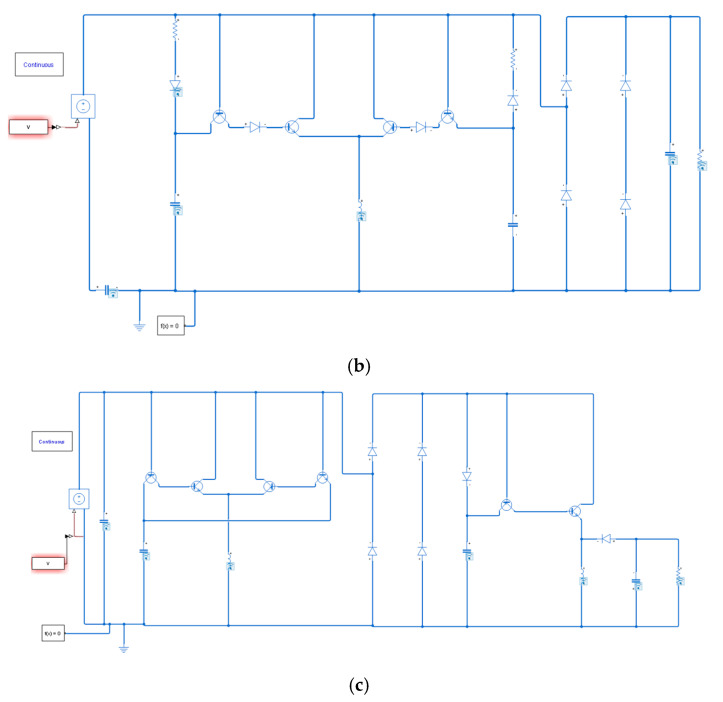
Three different energy-harvesting circuits. (**a**) Standard energy-harvesting circuit; (**b**) parallel-type synchronized switching circuit; (**c**) double synchronized switching (DSSH) circuit.

The energy-harvesting circuit introduced in Chapter 3 was modeled in Simulink (as shown in Figure 5), and the mechanical and electrical domains were solved simultaneously in MATLAB R2024a. The obtained open-circuit voltage and the voltage after connecting the circuit are illustrated in the figure.

**Figure 6:**
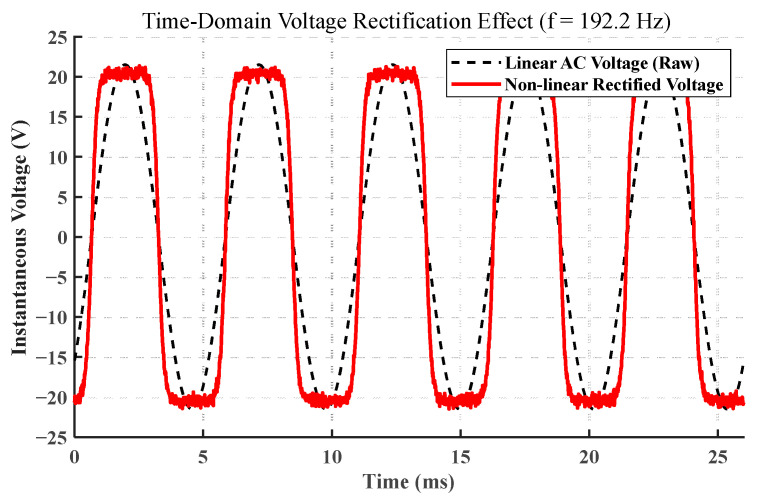
Open-circuit voltage and terminal voltage.

As shown in Figure 6, unlike the ideal harmonic AC voltage (black dashed line) observed under open-circuit or purely resistive conditions, the actual instantaneous voltage across the piezoelectric network (red solid line) exhibits a severe non-linear rectification effect. Once the voltage exceeds the rectification threshold, it is strictly clamped by the interface circuit, forming a pseudo-square waveform. This clamping period represents the effective energy-harvesting window, highlighting the necessity of employing non-linear electromechanical coupled models rather than simplified linear assumptions.

Since the inductor and capacitor in the circuit primarily serve to form an LC loop and store energy, they have minimal impact on the energy-harvesting power. Therefore, in this study, the capacitance is set to 5.1 μF and the inductance is set to 1 mH. On this basis, the load dependence of the circuit is investigated.

**Figure 7:**
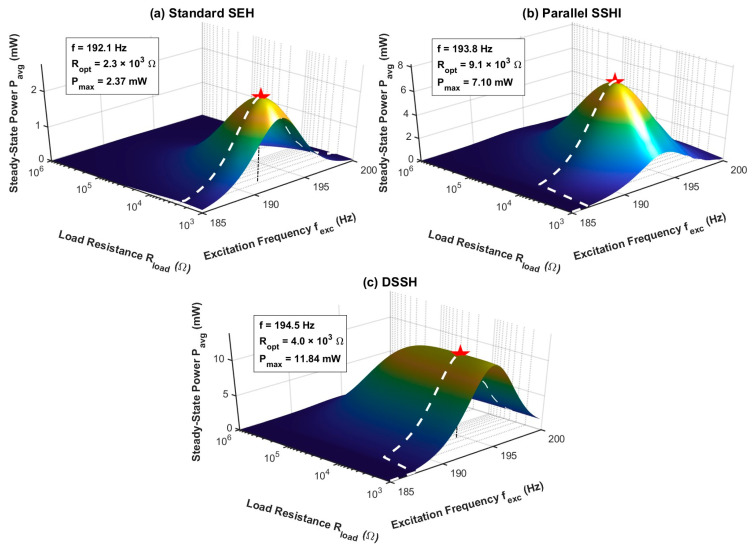
Illustrates the 3D coupled surfaces of steady-state harvested power concerning excitation frequency and load resistance. The white dashed line represents the optimal impedance matching trajectory across different excitation frequencies. The red star denotes the optimal power point achieved under optimal electro-mechanical resonance.

As shown in Figure 7, an analysis of the power amplitude reveals that the non-linear switching interfaces significantly outperform the standard SEH, which yields a peak power of 2.37 mW. Specifically, the parallel SSHI reaches 7.10 mW, whereas the DSSH topology achieves a maximum power of 11.84 mW. This nearly fivefold enhancement is primarily attributed to its superior synchronized voltage inversion and thorough energy extraction capabilities.

In addition to power amplification, a pronounced high-frequency shift in the resonance peak is observed, advancing from 192.1 Hz in SEH to 193.8 Hz in SSHI, and further to 194.5 Hz in DSSH. This spectral shift characterizes the equivalent stiffness enhancement effect induced by electromechanical coupling during non-linear switching actions; specifically, an intensified energy extraction process inherently introduces additional equivalent stiffness to the vibrating system. While this frequency shift in simulations is predominantly governed by the electromechanical coupling effect, additional factors contribute to this phenomenon in practical physical systems, including inherent structural non-linearities and dynamic variations in the elastic modulus of the piezoelectric material.

Furthermore, an evaluation of the surface topographies along the load resistance axis exposes critical differences in impedance matching. Unlike the SEH and Parallel SSHI configurations, which exhibit sharp power ridges indicative of high sensitivity to load variations, the DSSH topology demonstrates remarkable load-independent characteristics by forming a broad power plateau. This decoupling of the energy-harvesting process from the terminal load indicates that DSSH is not only highly efficient but also exceptionally robust under fluctuating environmental conditions.

## 5. Experimental Study on Piezoelectric Energy-Harvesting of the Blisk

### 5.1. Experimental Apparatus and Scheme Design

The proposed piezoelectric energy-harvesting scheme for the blisk uses the periodic vibration induced by external excitation or operating conditions as the energy source. Rectangular and circular PZT ceramic patches are bonded to regions with relatively high strain-energy density, such as the disk surface and the blade root, to enable the conversion of mechanical vibration energy into electrical energy. When the blisk vibrates at specific modes and resonant frequencies, bending deformation as well as tensile–compressive strain act on the PZT patches, generating an AC electrical signal whose frequency corresponds to the vibration frequency.

**Figure 8:**
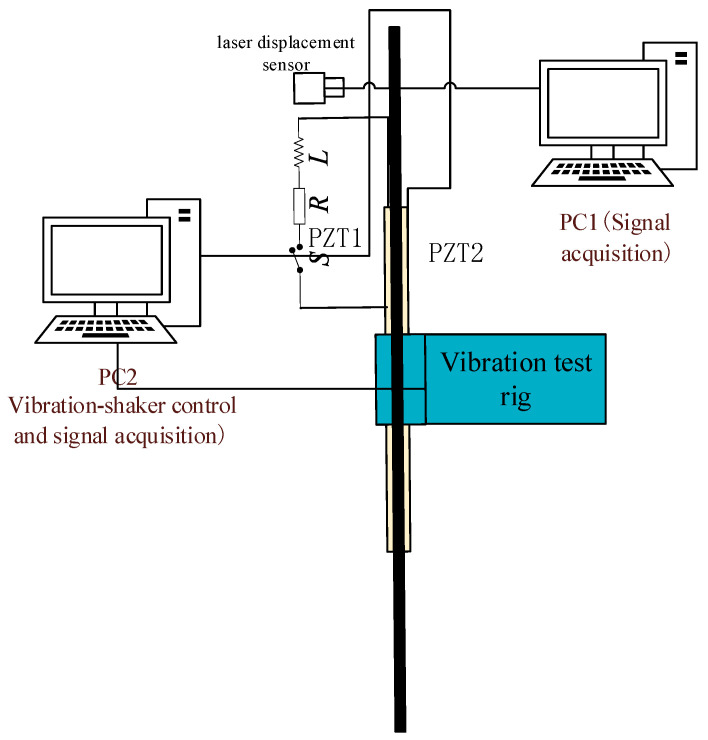
Schematic diagram of the piezoelectric bladed-disk experiment.

**Figure 9:**
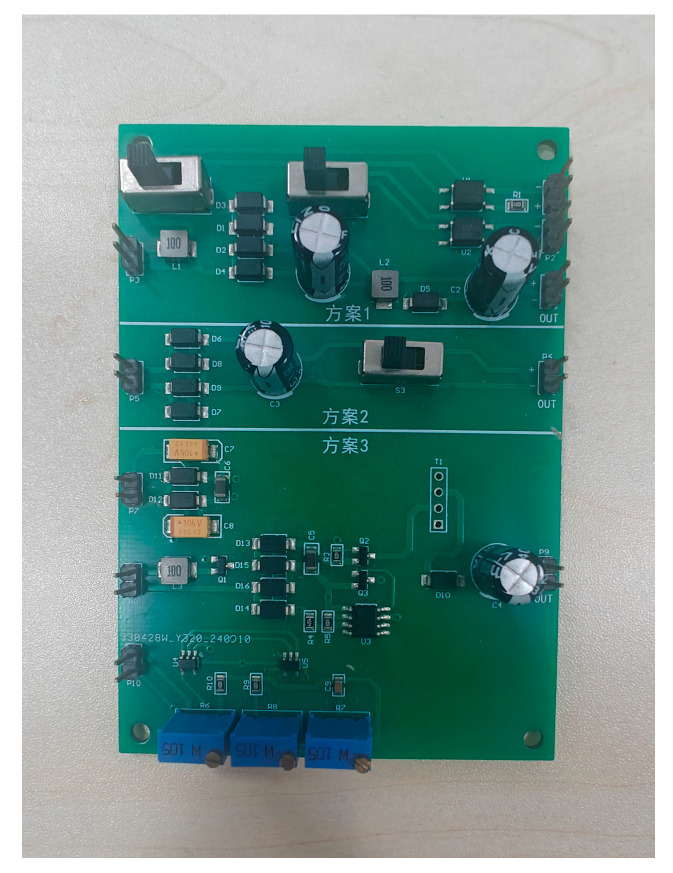
Photograph of the energy-harvesting circuit (prototype). The Chinese in the picture is Plan 1, Plan 2, Plan 3.

**Figure 10:**
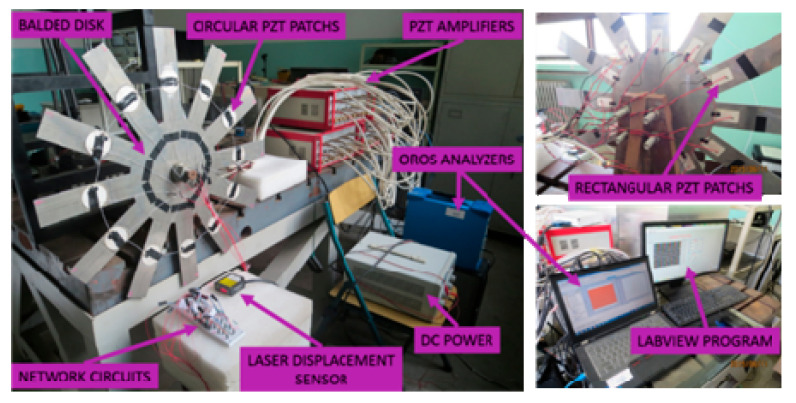
Photograph of the piezoelectric bladed-disk experimental setup.

The schematic diagram of the experimental plan, the energy harvesting circuit board, and the experimental physical objects are shown in Figure 8, Figure 9 and Figure 10.

To accommodate both energy-harvesting and measurement/diagnostic analysis, the electrical system adopts a parallel architecture with two independent branches, namely a measurement branch and an energy-harvesting branch. In the measurement branch, the signal is conditioned and amplified and then fed into an OROS analyzer and a LabVIEW platform for the synchronized acquisition of vibration and electrical signals, spectral analysis, frequency response function (FRF) identification, and modal characterization. In the energy-harvesting branch, the PZT output is processed by an energy-harvesting interface circuit for rectification, impedance matching, and filtering, and then stored in a storage capacitor or a supercapacitor, thereby producing a stable DC output to power an external load or low-power electronic devices.

During the experiments, a laser displacement sensor is employed to perform non-contact measurements of the vibration displacement at key locations of the blisk. A correlation analysis between the measured displacement and the PZT output signal is conducted to verify the correspondence between piezoelectric output and structural vibration, and to identify the most favorable operating modes and frequency bands for energy-harvesting. Furthermore, through frequency-sweep excitation and variable-load tests, the output voltage, current, and average power under different vibration states and electrical matching conditions are obtained, enabling determination of the optimal energy-harvesting performance of the system. The entire setup is powered by a DC supply for the amplifier, data acquisition, and control units, achieving a comprehensive validation of the energy-harvesting mechanism, efficiency, and engineering feasibility of the piezoelectric bladed-disk system, while ensuring that the measurement system remains stable and reliable without significantly degrading the harvesting performance.

In the experimental setup, the test structure is primarily made of 6061 aluminum alloy. The piezoelectric patches, made of PZT-5H with dimensions of 40 mm × 30 mm × 1 mm and a clamped capacitance of 68 nF, are bonded to the structure. A laser displacement sensor (Model: HL-G103-A-C5, Panasonic Industrial Devices SUNX Suzhou Co., Ltd., Suzhou, China) is employed to perform non-contact measurements of the vibration displacement at key locations of the blisk.

### 5.2. Energy-Harvesting Efficiency Analysis

**Figure 11:**
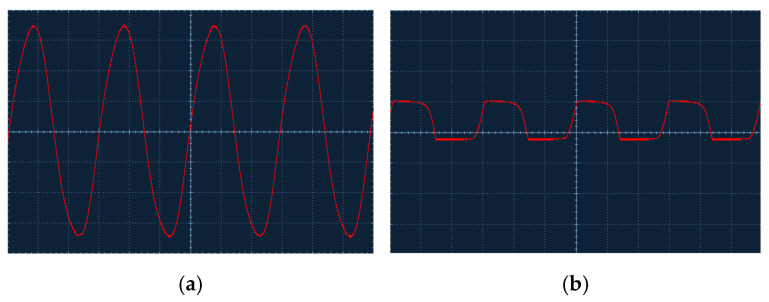
Oscilloscope waveforms before and after connecting the SSDI circuit. (**a**) Open-circuit voltage across the piezoelectric patch, (**b**) Square-wave voltages across the piezoelectric patches after connecting to the SSDI.

To compare the operating characteristics of the SSHI interface, the voltage waveforms generated by the piezoelectric patch before and after connecting the SSHI circuit are examined. The voltage waveforms and the corresponding comparative voltage-response plots are shown in Figure 11.

As shown in Figure 11, within the selected sampling interval under harmonic excitation of the cantilever beam, when the piezoelectric patch operates under open-circuit conditions, the generated voltage is proportional to the vibration displacement and exhibits a sinusoidal waveform. After the SSHI interface is connected, the voltage response across the piezoelectric patch decreases, and the waveform becomes approximately square-shaped. This indicates that, after connecting the SSHI circuit, the switching instants coincide with the moments when the structural displacement reaches its extrema, which is consistent with the operating principle of SSHI and confirms that the intended design objective is achieved.

**Figure 12:**
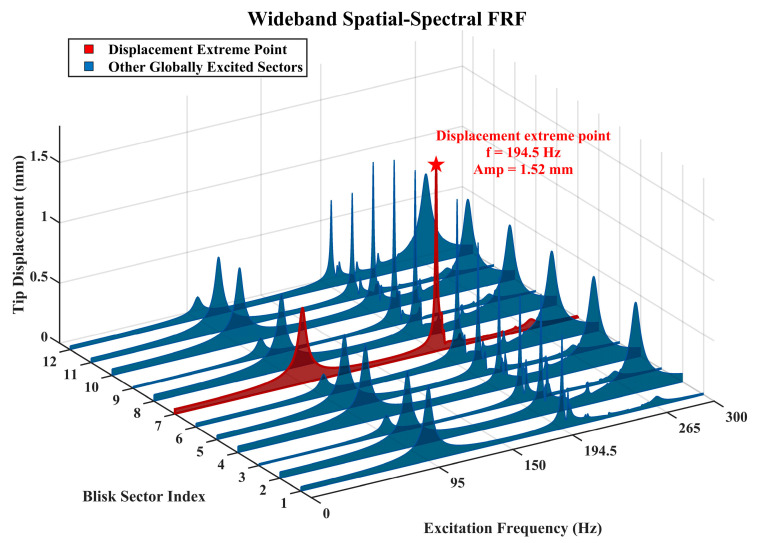
Swept-frequency displacement plot of the bladed disk.

A frequency-domain analysis of the voltage response in each branch reveals that the system exhibits random perturbations, with the maximum peak occurring at 194.5 Hz, as shown in Figure 12.

To comprehensively understand the dynamic behavior of the entire blisk, a wideband frequency sweep was conducted under global uniform excitation. Figure 12 illustrates the resulting spatial–spectral frequency response across all 12 sectors. The experimental emulation explicitly captures multiple modal families. While the low-frequency modes and high-frequency modes exhibit relatively distributed and moderate energy profiles, a distinct phenomenon occurs in the mid-frequency region.

Due to the inherent structural mistuning, the cyclic symmetry of the blisk is severely broken. At exactly 194.5 Hz, the vibration energy is drastically funneled and confined into a single sector (highlighted in red). This fundamental dynamic analysis physically justifies that deploying the non-linear harvesting interface directly on this localized sector will yield the maximum energy extraction.

Having identified the optimal mechanical hotspot, a detailed impedance matching analysis was subsequently performed. The excitation frequency was strictly locked at the 194.5 Hz hotspot, and the terminal load resistance was systematically swept from $10^{3}$ to $10^{6}$ Ω.

**Figure 13:**
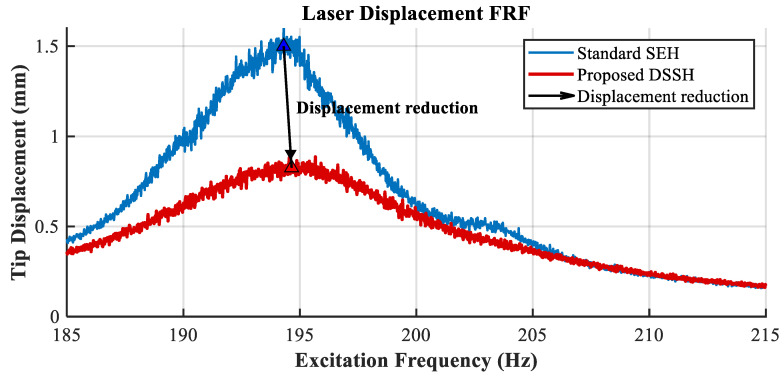
Comparison of tip displacement response between standard SEH and proposed DSSH. The triangular markers in the figure stand for extreme values of displacement.

Figure 13 compares the displacement responses of the blisk with the DSSH circuit and the SEH circuit connected. It can be observed that the DSSH circuit achieves better vibration reduction and energy conversion performance than the SEH circuit.

**Figure 14:**
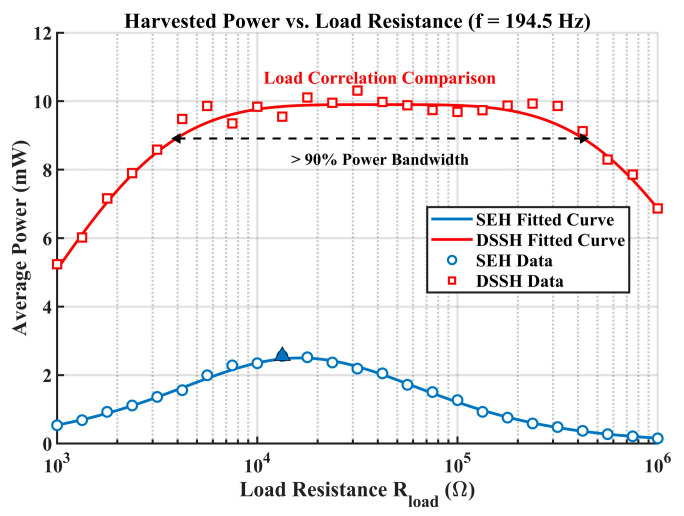
Harvested power comparison between standard SEH and proposed DSSH. The blue triangular symbol represents the optimal resistance value.

Figure 14 compares the average harvested power of the standard SEH and the proposed DSSH topologies, demonstrating excellent agreement between the theoretical models (solid lines) and the experimental data points (scatter markers).

As clearly observed, the standard SEH (blue curve) exhibits a highly sensitive, sharp power peak. It yields a maximum power of merely 2.56 mW strictly at an optimal resistance of 13.3 kΩ, beyond which the performance deteriorates precipitously. In stark contrast, the DSSH interface (red curve) not only massively amplifies the peak power to approximately 10 mW (a nearly 4-fold enhancement) but also demonstrates a remarkable load-independent plateau. Because the intermediate inductor in the DSSH circuit decouples the piezoelectric capacitance from the terminal load, it maintains a >90% power bandwidth across an extremely broad resistance range. This superior energy decoupling capability proves that the DSSH topology is highly resilient to electrical impedance fluctuations, making it exceptionally suitable for the complex operating environments of mistuned blisk systems.

## 6. Conclusions

To address the self-powering requirements of aero-engine blisks, this paper investigates the integration of the structural mistuning-induced vibration localization effect with nonlinear synchronized switch energy-harvesting technology. Through theoretical modeling, simulation, and experimental validation, the dynamic response and electrical performance of different interface circuits are characterized. The main conclusions are as follows:

(1) Breaking the conventional approach that treats mistuning solely as a negative factor, the positive gain phenomenon of the mistuning localization effect on piezoelectric energy-harvesting is elucidated. Analysis of the nine-sector blisk model indicates that structural stiffness mistuning disrupts cyclic symmetry, leading to frequency splitting and spatial energy redistribution. Near 194.5 Hz, the vibration energy concentrates in a specific sector (with the amplitude amplified by 6.35%). Deploying piezoelectric transducers within these localized zones provides a structural basis for improving harvesting efficiency.

(2) The energy conversion characteristics of synchronized switch circuits and the electromechanical equivalent stiffness strengthening phenomenon are observed. Unlike the standard energy-harvesting (SEH) circuit, the P-SSHI and DSSH circuits achieve voltage polarity reversal at displacement extrema, reducing capacitive losses. Furthermore, as the nonlinear circuits extract energy, the overall equivalent stiffness of the system is modified. This manifests as a rightward frequency shift in the resonance peak (from 192.1 Hz for SEH to 194.5 Hz for DSSH), reflecting the equivalent stiffness strengthening phenomenon induced by electromechanical coupling.

(3) Based on the simulation and swept-frequency test data, under excitation at the localized resonance frequency (194.5 Hz), the SEH circuit yields a maximum average power of 2.56 mW. The P-SSHI circuit increases the power to 7.10 mW, while the DSSH circuit achieves a maximum of approximately 10 mW. This demonstrates a nearly 4-fold power enhancement compared to the standard circuit.

(4) The SEH and P-SSHI circuits exhibit load dependency, and their power output decreases when the load deviates from the optimal impedance. Benefiting from the internal energy storage inductor that decouples the electromechanical energy from the terminal load, the DSSH topology maintains a load-independent plateau (>90% peak power) across a wide resistance range. This resilience to electrical parameter variations indicates that the DSSH topology is a practical choice for self-powered blisk systems under varying operating conditions.

## Figures and Tables

**Table 1:** Lumped parameters of the electro-mechanically coupled bladed disk.

Blade tip mass ($m_{1}$)	0.25	Blade root mass ($m_{2}$)	0.35
Disk mass ($m_{d}$)	1.2	Blade tip stiffness ($k_{1}$)	2 × 10 ^6^
Blade root stiffness ($k_{2}$)	1 × 10 ^6^	Disk stiffness ($k_{d}$)	5 × 10 ^7^
Piezoelectric patch capacitance	50 nF	Electromechanical coupling coefficient	0.2
inductance value	10 mH	The filter capacitor	1 nF
Energy storage capacitor	5 μF	Diode forward voltage	0.7 V

**Table 2:** Vibration amplitudes of the tuned blisk sectors.

Blade Index	Tuned Resonance Freq/Hz	Tuned Max Amplitude/mm
1	192.19	0.53611
2	192.19	0.41068
3	192.19	0.093095
4	192.19	0.26806
5	192.19	0.50378
6	192.19	0.50378
7	192.19	0.26806
8	192.19	0.093095
9	192.19	0.41068

**Table 3:** Vibration amplitudes of the mistuned blisk sectors.

Blade Index	Mistuned Resonance Freq/Hz	Mistuned Max Amplitude/mm
1	191.99	0.57019
2	191.95	0.36118
3	191.91	0.09955
4	192.02	0.21825
5	192.01	0.53509
6	191.97	0.49499
7	191.95	0.29606
8	192.17	0.097065
9	192.01	0.39666

## Data Availability

Data are contained within the article.
